# Physiological and psychosocial stressors among hemodialysis patients in the Buea Regional Hospital, Cameroon

**DOI:** 10.11604/pamj.2018.30.49.15180

**Published:** 2018-05-18

**Authors:** Odette Dorcas Manigoue Tchape, Youth Brittany Tchapoga, Catherine Atuhaire, Gunilla Priebe, Samuel Nambile Cumber

**Affiliations:** 1 Faculty of Health Sciences, Department of Nursing, University of Buea, Cameroon; 2 School of Health and Human Services, Saint Monica University, Cameroon; 3 Faculty of Medicine, Department of Nursing, Mbarara University of Science and Technology, Uganda; 4 Section for Epidemiology and Social Medicine, Department of Public Health, Institute of Medicine (EPSO), The Sahlgrenska Academy at University of Gothenburg, Gothenburg, Sweden

**Keywords:** Physiological stressor, psychosocial stressor, hemodialysis, patients, Cameroon

## Abstract

**Introduction:**

End Stage Renal Disease (ESRD) is an irreversible kidney condition and hemodialysis is the most frequent treatment option used for this condition. However, hemodialysis also has a detrimental impact on the quality of life and the individuals' physical and psychosocial wellbeing. The main objective of this study was to identify physiological and psychosocial stressors faced by patients undergoing hemodialysis in the Buea Regional Hospital in Cameroon.

**Methods:**

A cross-sectional study was carried out (December 2016 - January 2017) among patients undergoing hemodialysis at the Buea regional hospital. Data were collected with the use of a structured questionnaire and analyzed using SPSS version 21.0. Quantitative variables were expressed as frequencies, percentages and means.

**Results:**

Among the patients undergoing hemodialysis, 28 (70.0%) were below 5-year dialysis while 12 (30.0%) had been on dialysis for five years and more. 21(52.5%) were male and 19(47.5%) female. Half of the patients were married 20(50%), 13(32.5%) were single, 6(15%) were divorced, and one (2.5%) was a widower. Also, 28 (70.0%) were below 5-year dialysis while 12 (30.0%) had been on dialysis for five years and more. All participants experienced at least one or more physiological and psychosocial stressors. Among physiological stressors, the most frequent were feeling tired (97%), followed by arterial and venous stick (88%) while itching (49.5%) was the least noted physiological stressor. Among psycho-social stressors, the most recurrent were transportation to and from the hospital (99.5%), cost of treatment (99.5%) and Limits on time and place of vacation (99%), followed by Limitation in physical activities, frequent hospitalizations, the length of time on dialysis, uncertainty about the future, changes in life style, increased dependence and sleep disturbances.

**Conclusion:**

The topic of stressors is of importance among patients receiving dialysis, as these affect their psycho-social and physiological wellbeing. Thus, nephrologists, nurses and family members play an important role in providing patients with effective psycho-social and physiological support.

## Introduction

Chronic kidney disease (CKD) is a global public health problem of great concern to Sub-Saharan Africa (SSA) [1]. It is progressive in nature and the last stage is called end-stage renal disease (ESRD). In 2010, chronic kidney disease was classified as the 18^th^ cause of death worldwide and the prevalence is said to have doubled between the year 1990 and 2010 [2]. It is estimated that over the next decades, more than 70% of patients with ESRD will be residents in low-income countries. The total prevalence of chronic kidney disease in Cameroon is unknown, yet prevalence in the population of the Western region of Cameroon is estimated to be 13.2% [3]. The severity of kidney disease is determined by the individual's glomerular filtration rate (GFR), which is the best overall index of kidney function in health and disease [4]. A glomerular filtration rate of 90 mL/minute per 1.73m^2^ or higher is considered normal. A decreasing GFR indicates increasing kidney impairment. A glomerular filtration rate less than 60 mL/minute per 1.73m^2^ indicates a loss of approximately half the kidney's normal function in an adult. As the GFR continues to decline, occurrence of complications related to kidney disease, such as anemia, bone disease and malnutrition, increase [5, 6]. It has been called “Africa's forgotten disease” as the health care system in many countries has not had the resources for treatment [7]. The management of advanced chronic kidney disease is renal replacement therapy (RRT) which includes renal transplantation, hemodialysis and peritoneal dialysis. These remedies are typically unavailable, cost intensive and therefore inaccessible to many affected individuals in Sub-Saharan Africa. Moreover, shortage of skilled personnel is responsible for the high rates of morbidity and mortality [8]. Hemodialysis does not cure ESRD, it keeps the patient alive. Nonetheless these sustainable therapies negatively affect patients' quality of life both physically and mentally since they must adjust to a new lifestyle. Also, majority of patients find it thorny to accept new image, habits, complete dependence on the machine for survival, changes in their physical health, limited activities, rigorous treatment plan and dietary restrictions. Eventually, their functional status, personal relationship, social and economic status are affected considerably [9-11]. Patients with ESRD experience different levels of discomfort in response to various types of physiological and psychosocial stressors. However, coping strategies can affect morbidity or mortality. Moreover, what kind of coping strategies patients use depends on their personal experience, social support system, individual beliefs and availability of resources. In Cameroon, there are-to our knowledge-no studies related to physiological and psychosocial stressors in hemodialysis patients despite the increase in the number of patients receiving hemodialysis. Therefore, identifying such stressors is a crucial step to improve the quality of health care offered to these patients. Hence, this study sought to identify the physiological and psychosocial stressors experienced by patients undergoing hemodialysis.

## Methods

### Definition of terms

**Stressor**: A chemical or biological agent, environmental condition, external stimulus or an event that causes stress to an organism.

**Physiological stressors**: External or internal conditions that challenge the functioning of a cell or an organism. It is also the presence of the stressor, followed by the sending of signals to the brain, and to the specific sympathetic and hormonal responses to eliminate, reduce or cope with the stress.

**Psychosocial stressors**: Social events and situations that, in the individual's cognitive interpretation, threatens to require additional, possibly non-available, resources.

**Study design and setting**: A descriptive cross sectional study was carried out from December 2016 - January 2017 in the Buea Health District. Cameroon has 10 regions with 9 dialysis centres. The South West Region has only one dialysis Centre found at the Buea Regional hospital and it is meant to cover a population of 1,384,286 inhabitants. The BRH serves clients from the greater Buea area. Most of the patients with ESRD undergo at least 3 dialysis sessions per week which is cost intensive (5,000 FCFA per session) and therefore inaccessible by many affected individuals. This could be the reason for low registration of patients.

**Participants**: The sample included 55 patients undergoing in-center hemodialysis at the Regional Hospital Buea.

**Inclusion criteria**: Diagnosis of ESRD; current hemodialysis; age 18-65 years; English or French speaking; volunteer participation and signed consent form.

**Exclusion criteria** Use of anti-stress and anxiolytic drugs; and individuals who had been on dialysis for less than 2 months.

**Sampling technique**: Non-probability convenience sampling was thus utilized as the 55 patients that met the inclusion criteria and were undergoing hemodialysis at the Buea regional hospital during the study period, received information about the study. Out of these 40 patients accepted participation [12].

**Data collection instrument**: A self-administered questionnaire was used to collect data on physiological and psychosocial stressors among hemodialysis patients. The questionnaire was divided into two sections. The first section was based on demographic profiles of respondent and the number of dialysis session per week. Section two consisted of Hemodialysis Stress Scale (HSS) questionnaire [13] which contained questions pertaining to both physiological and psychosocial stressors. Patients were asked to rate the extent they were troubled by each one of the psychological concerns on a 5-point Likert scale from 1 (*strongly disagree*) to 5 (*strongly agree*), where higher numbers indicated greater severity of the impact of each factor on these patients.

**Ethical considerations**: Ethical clearance was obtained from the institutional review board of the Faculty of Health Sciences, University of Buea, Cameroon (2016/104/UB/SG/IRB/FHS). Administrative approval was obtained from the South West Regional Delegation of Public health and the director of the Buea Regional Hospital Buea, Cameroon. An informed written consent form was provided to each participant after briefing them about the procedure of the study and its objectives. The participants were informed of the general nature of the study as well as the implications of participation. They were assured of confidentiality and they were also told that they are free to decline or discontinue participation in the study.

**Data analysis**: After data collection and cleaning, data were captured in Microsoft Excel (2010) spreadsheet and imported to SPSS statistical package version 20.0 for analysis. Descriptive analysis was carried out by calculating the mean, median, standard deviation and frequencies of different variables using the SPSS v 16.0. The prevalence rates of Hepatitis B virus were determined by simple percentages and summarized in tables.

## Results

Among the patients undergoing hemodialysis, 21 (52.5%) were male and 19 (47.5%) female. The minimum and maximum age was 20 and 70, respectively. Half of the patients were married 20 (50%), 13 (32.5%) were single, 6 (15%) were divorced and one (2.5%) was a widower. Also, 28 (70.0%) were below 5-year dialysis while 12 (30.0%) had been on dialysis for five years and more (Table 1).

**Table 1:** Demographic characteristics of patients

Variables	Frequency (N)	Percentage (%)
**Gender**		
Male	21	52.5
Female	19	47.5
**Age**		
20-29 Years	8	20.0
30-39 Years	10	25.0
40-49 Years	7	17.5
50-60 Years	7	17.5
61-65 Years	8	20.0
**Marital Status**		
Single	13	32.5
Married	20	50.0
Divorced	6	15.0
Widow/Widower	1	2.5
**Occupation**		
Unskilled	12	30
Skilled Workers	4	10
Government Employees	6	15
Business	4	10
Others	14	35
**Dialysis History**		
Below 5 years	28	70.0
≥ 5 years	12	30

**Physiological stressors**: Among physiological stressors, the most frequent were feeling tired (97%), followed by Arterial and venous stick (88%) while itching (49.5%) was the least reported as shown on (Table 2).

**Table 2:** Physiological stressors in hemodialysis patient

Physiological stressors	Percentage (%)
Feeling tired	97
Arterial and venous stick	88
Loss of body functions	82
Stiffening of joints	73.5
Muscle cramps/soreness	62.5
Nausea and vomiting	51
Itching	49.5

**Psychosocial stressors**: Among Psycho-social stressors, the most recurrent were transportation to and from the hospital (99.5%), cost of treatment (99.5%) and limits on time and place of vacation (99%). The least recurrent was decreased ability to have children (51%) as shown on (Table 3).

**Table 3:** Psychosocial stressors in hemodialysis patient

Psychosocial stressors	Percentage (%)
Transportation to and from the hospital	99.5
Cost of treatment/ transportation/ or other costs	99.5
Limits on time and place of vacation	99
Limited in style of clotting	97.5
Limitation in physical activities	97
Decrease in social life	96
Changes in body appearance	95
Limitation in fluid	94.5
Decrease in sexual derive	94.5
Limitation in food	94
Interference with job	94
Length of treatment	90
Feelings related to treatment (feeling cold)	89
Uncertainty about the future	88
Boredom	85
Fear of being alone	84
Dependency on physicians	83.5
Dependency on nurses and technicians	81.5
Frequent hospital admissions	81.5
Sleep disturbances	80.5
Changes in family responsibilities	80
Reversal in role with spouse	75.5
Reversal in role with children	73
Dialysis machine/ or equipment	57
Decreased ability to have children	51

## Discussion

This study was carried out to identify stressors faced by patients undergoing hemodialysis in BRH. This study in Beua, Cameroon, confirms what studies in other settings have shown: hemodialysis is strongly associated with physiological and psychosocial stressors. Moreover, this study indicates an even stronger association as more than 70% of the participants noted 25 out of 29 stressors, which was at the higher end in comparable studies carried out on patients that had been in treatment for a longer period [13] compared to the present study. In relation to physiological stressor, the participants noted primarily the stressors feeling tired, arterial and venous stick and Loss of body functions. Yet, even the stressor that was noted to a lower degree (stiffening of joints, muscle cramps, nausea and vomiting and itching) were noted by approximately 50-70% of the participants. The significant relationship between the duration of treatment and the level of fatigue is in line with similar studies. The experience of fatigue has been more commonly reported by respondents who had been receiving treatment for more than 2 years, compared to those treated for less than 2 years [14-21]. This indicates that the notes on fatigue as a stressor would have been even higher if the present study population had been in treatment for longer period. Fatigue is a subjective symptom illustrated by tiredness, weakness and lack of energy, yet one of the most debilitating symptom reported by hemodialysis patients [14]. Factors that may contribute to fatigue in dialysis patients includes anemia, malnutrition, inflammation, creatinine and albumin levels, depression, and sleep disorders [18, 22-24]. Anemia resulting from reduced erythropoietin production has been cited as an important cause of fatigue in this population. Additionally, patients undergoing chronic HD show evidence of accelerated protein catabolism, which might be due to the significant loss of amino acids induced by dialysis [25]. Thus, it is reasonable to presume that lower levels of albumin can be significantly correlated with greater levels of fatigue [24-26]. Socio-demographic factors, including age, sex, race, educational, marital, and employment status may also play a role in experiencing fatigue in dialysis patients [27-29]. This together with study results indicates that the risk of fatigue should be an integrated aspect of care for dialysis patients.

The second most reported physiological stressor was Arterial and venous stick, which is higher compared to similar studies. A qualitative study of Finnegan-John [30] reported that patients with ESRD receiving HD developed a new identity and sense of self, as patients might perceive themselves as unattractive. Hemodialysis vascular access via a fistula, neck line, or catheter altered the appearance of the body as many patients communicated feelings of discomfort in exposing their fistula and hide it under clothing. Bamgboye [31] in a study on hemodialysis management problems in developing countries noted that the shortage of skilled technicians and surgeons in the creation of arteriovenous fistula (AVF) access has led to failure in arteriovenous fistula which alters body appearance. In line with findings in another west African study [32] as well as in a recent systematic review 49.5% reported itching or pruritus as a physiological stressor. Pruritus is an unpleasant cutaneous sensation that arouses the urge to scratch. Its pathophysiology is likely multifactorial and the effectiveness of available treatment is uncertain [33, 34].

**Psycho-social stressors**: Many of the noted psychosocial stressors are associated with the logistics and economic aspects of being in treatment. Transportation; cost of treatment/transportation/or other costs; decrease in social life; interference with job; length of treatment were all reported by more than 90% of the participants. According to studies conducted by Tsay [35] and Finnegan-John [30], length of dialysis treatment and transportation difficulties were reported as stressors for the participants. This is because patients receiving hemodialysis had to devote 4 hours for each dialysis session three times a week. In developing countries, most hemodialysis centres are still located in the big cities and as such patients travel long distances to reach the hemodialysis centre coupled with the time-consuming nature of the treatment [31]. In contrast to our findings, Gorji [36] noted that only 45% reported travelling related worry and 41.5 % had cost related worries. This difference may be due to poor health infrastructure as well as difficult accessibility to health facilities in Cameroon. Moreover, most Cameroonians do not have the financial means to sustain three dialysis sessions per week despite their monthly income. It is worth noting that the GDP of Cameroon was 1357,1 USD (678,550 FCFA) [37] at the time of the study and three session per week cost (40 USD) 5000FCFA leaving the patient with little or nothing to cater for their needs. Also, many hemodialysis patients are even unemployed and reason why most appear in the hospital at a critical stage for consultation.

Adherence to dietary regimen and fluid limitation is a persistent challenge to HD patients due to constant choices about food and drink, existing cultural practices, and related illnesses [38-41]. Dietary adaptation is essential to cut down the risk of morbidity and mortality in patients undergoing dialysis. Moreover, changes in the intake of energy, macronutrients, certain minerals and fluid are also crucial for achieving good therapeutic results and better outcomes [42-44]. Hong [39] states that dietary constrain for hemodialysis patient is an erroneous practice that may aggravate hypoglycemia and nutritional derangements. Statistics have shown that the prevalence of non- adherence to diet and fluid is 81.4% and 74.6% respectively [45]. It should be emphasized that compliance with fluid restriction is problematic during hot periods of the year, when patients have an increased thirst. On the other hand, Stavroula [10] demonstrated that restriction of fluid, dependence on dialysis machine and/or equipment, limitation of physical activity, changes in family responsibilities, dependency on nurses and technicians, length of treatment, dependency on physicians, sleep disturbance and limitation of food were the most prevalent stress sources among these patients.

## Conclusion

The influence of the symptoms of End Stage Renal Disease on patients' quality of life, and the frequency of periodic hemodialysis, the compliance of patients with treatment regimen and the negative side effects of the disease on patients is the strongest Stressors, which significantly affect the physiological and Psycho-social health of the patient. Physiological and psychosocial stressors have not been weighed against each other in this study, yet compared to other studies the psychosocial stressors seem to be particularly difficult to cope with for the participants in this study. This may partly be connected to the Cameroonian context with its constrained health care infrastructure, few dialysis centers, limitations in public transportation and social security system, etc. which increases logistical problems as well as social and economic pressure on the patient. Therefore, treatment regimens for patients undergoing hemodialysis in Buea Regional Hospital should include broad strategies and activities for preventing psychosocial and physiological stressors, e.g psychological counseling, health education and treatment for physiological stressors.

### What is known about this topic

- Hemodialysis is a lifesaving treatment for patients with End-Stage Renal Disease, yet it has adverse physiological and psychosocial effects;
- If patients are physiologically and psychosocially stressed, they may be less compliant with their treatment regimens;
- Haemodialysis itself is a psycho-social stressor to these patients.

### What this study adds

- There is need for a comprehensive health care plan for patients with End-Stage Renal Disease including psychological counseling and other support services connected to the dialysis center
- Health education specifically focusing on common stressors as well as factors that are essential for efficacious treatment should be mandatory for patients undergoing hemodialysis;
- The establishment of additional dialysis centers should be considered by health authorities in order to facilitate treatment compliance and reduce psychosocial stressors connected to logistical, social and economic difficulties.

## Competing interests

The authors declare no competing interest.
